# Distinguishing contributions of ceramic matrix and binder metal to the plasticity of nanocrystalline cermets

**DOI:** 10.1107/S2052252519015471

**Published:** 2020-01-01

**Authors:** Xinru Ge, Xuemei Liu, Chao Hou, Hao Lu, Fawei Tang, Xiangfei Meng, Wenwu Xu, Xiaoyan Song

**Affiliations:** a College of Materials Science and Engineering, Key Laboratory of Advanced Functional Materials, Education Ministry of China, Beijing University of Technology, Beijing 100124, People’s Republic of China; b National Supercomputer Center in Tianjin, Tianjin 300457, People’s Republic of China; c Department of Mechanical Engineering, San Diego State University, San Diego, CA 92182, USA

**Keywords:** nanocrystalline cermets, dislocation interactions, plastic deformation, molecular dynamics simulations, mechanical properties, composite materials, computational modeling, dynamical simulations, materials modeling, nanostructures

## Abstract

Using the typical WC–Co cemented carbide as an example, the interactions of dislocations within the ceramic matrix and the binder metal, as well as the possible cooperation and competition between the matrix and binder during deformation of the nanocrystalline cermets, were studied by molecular dynamics simulations. It was found that at the same level of strain, the dislocations in Co have more complex configurations in the cermet with higher Co content. With loading, the ratio between mobile and sessile dislocations in Co becomes stable earlier in the high-Co cermet. The strain threshold for the nucleation of dislocations in WC increases with Co content. At the later stage of deformation, the growth rate of WC dislocation density increases more rapidly in the cermet with lower Co content, which exhibits an opposite tendency compared with Co dislocation density. The relative contribution of Co and WC to the plasticity of the cermet varies in the deformation process. With a low Co content, the density of WC dislocations becomes higher than that of Co dislocations at larger strains, indicating that WC may contribute more than Co to the plasticity of the nanocrystalline cermet at the final deformation stage. The findings in the present work will be applicable to a large variety of ceramic–metal composite materials.

## Abstract 2

Contributions to plasticity from hard matrix and binder metal in nanocrystalline cermets were studied by molecular dynamics simulations.

## 1. Introduction   

The WC–Co cemented carbides are typical cermet materials whose hardness and wear resistance are attributed to the hard phase of WC and the toughness mainly stems from the Co binder phase (Ortner *et al.*, 2014 ▸). In addition to the high hardness and wear resistance, good toughness is also strongly demanded for the cermets that are widely applied as mining tools, molds and impact drills in various industry fields (Ashby *et al.*, 1989 ▸; Evans & McMeeking, 1986 ▸; Exner, 1979 ▸; Riesch *et al.*, 2013 ▸; Sigl & Fischmeister, 1988 ▸). It is generally considered that the plasticity and toughness of the cermets are determined by the binder metal due to its intrinsic mechanical features (García *et al.*, 2018 ▸). However, in our previous work, we found that the carbide matrix is also resistant to transgranular fracture through its deformation behavior (Liu *et al.*, 2015 ▸). Therefore, to distinguish the contributions of carbide matrix and binder to the plasticity of cermets is of great importance (Fischmeister *et al.*, 1988 ▸; Sigl *et al.*, 1988 ▸; Sigl & Schmauder, 1988 ▸).

A number of researchers have reported that in the binder phase of the composites, mechanical twinning, planar slip and phase transformation can occur during the process of deformation (Erling *et al.*, 2000 ▸; Roebuck *et al.*, 1984 ▸; Takahashi & Friese, 1965 ▸; Vasel *et al.*, 1985 ▸). Roa *et al.* studied the effects of microstructures on the flow stress of constrained metallic binder in cemented carbides with different compositions (Roa *et al.*, 2016 ▸). Petisme *et al.* investigated the influence of the thickness of the binder phase on the slip of WC/Co phase boundaries by molecular dynamics (MD) simulations (Petisme *et al.*, 2015 ▸). In our previous work, we examined the effect of WC/Co interfacial features on the deformation behavior of the cemented carbides (Feng *et al.*, 2017 ▸; Liu *et al.*, 2018 ▸, 2019 ▸; Xie *et al.*, 2016 ▸) and found that the coherent or semi-coherent WC/Co phase boundaries are beneficial for the increase of toughness of the nanocrystalline cemented carbides. However, to date there have been limited reports on the contribution of WC to the plastic deformation of nanocrystalline cermets, particularly on the distinction of the relative contributions of WC and Co at different deformation stages. Moreover, detailed and quantitative studies on the possible origins and mechanisms for the plastic deformation in the cermets on the nanoscale are required. Based on the above considerations, our present work is therefore focused on the possible cooperation and competition between WC and Co phases during deformation of the nanocrystalline cermets, particularly the interactions of dislocations within WC and Co in the cermets with different Co contents.

## 2. Methods   

**Figure 1:**
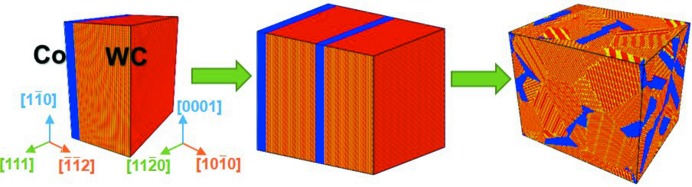
Simulation model of the nanocrystalline WC–Co cermet bulk material.

In this study, three kinds of nanocrystalline WC–Co bulk samples with Co contents of 8, 12 and 16 wt% were generated by the Voronoi construction method (Voronoi, 1908 ▸). The simulation models for the bulk material are shown in Fig. 1 ▸ and the related parameters are listed in Table 1 ▸.

Periodic boundary conditions were applied in all directions of the simulated WC–Co bulk materials. Firstly, the nanocrystalline cermet models were equilibrated at 300 K for 50 ps using a Nose/Hoover-type equation of motion sampled from the isothermal–isobaric (NPT) ensemble. Then, the uniaxial compression was performed along the *z* axis for each material at 300 K and at a constant strain rate. The stress was applied by continuously decreasing the height of the simulated sample and remapping all the coordinates of the atoms to the new positions at each time step. This process continued until the occurrence of the microcrack in the simulated material. The pressure was kept at zero in the other two directions.

The MD simulations were performed using the *Large-scale Atomic/Molecular Massively Parallel Simulator* (*LAMMPS*) (Plimpton, 1995 ▸) open-source code. We used the analytical bond order potential (ABOP) for the WC–Co cermet, which was developed by first-principle calculations and experimental data and has been successfully applied in previous studies (Albe *et al.*, 2002 ▸; Björkas *et al.*, 2009 ▸; Erhart & Albe, 2005 ▸; Li *et al.*, 2011 ▸). The atoms were colored according to their local structural environment using the polyhedral template matching (PTM) method (Larsen *et al.*, 2016 ▸) in the *OVITO* software (Stukowski, 2009 ▸), which can identify the grain boundaries and dislocations. The dislocation extraction algorithm (DXA) (Stukowski & Albe, 2010 ▸) in *OVITO* was used to distinguish the types of dislocations and calculate the length of the dislocation lines. The dislocation density was calculated by the total length of the dislocation lines per unit volume.

## 3. Results   

### 3.1. Stress–strain behavior   

**Figure 2:**
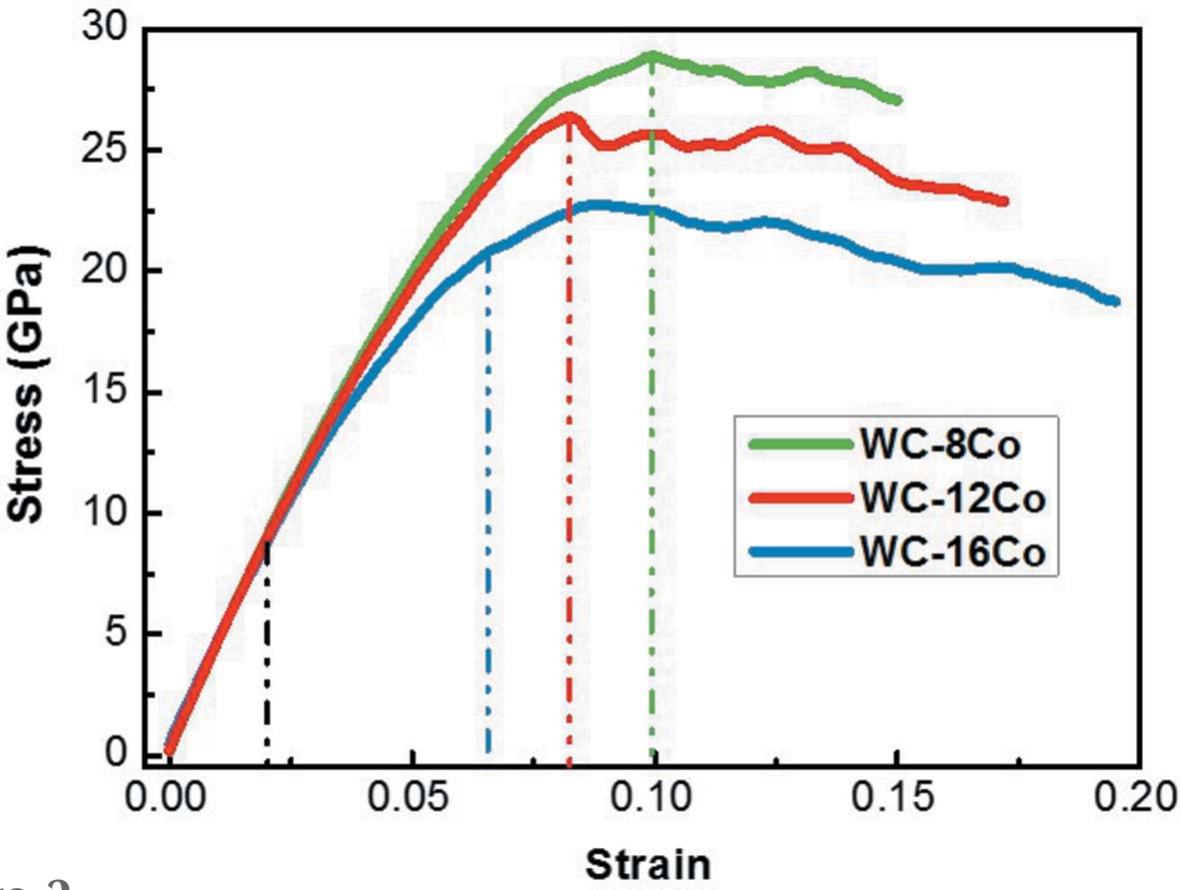
Stress–strain curves of the nanocrystalline WC—8Co, WC—12Co and WC—16Co cermets under uniaxial compression. Yield points are indicated by the dotted lines for each material.

Fig. 2 ▸ shows the stress–strain curves of the nanocrystalline WC–8Co, WC–12Co and WC–16Co cermets under uniaxial compression. It should be noted that the strains in the simulations are obviously larger than those that can be achieved in practice for the ordinary WC–Co cermets. This is due to the intrinsic feature of the MD method where the simulated strain values are not comparable to real strains. As the same strain rate was applied for all the simulated samples, the strains in the simulations are comparable for different samples, which reflects the difference in the deformation states of the samples.

The stress–strain curves can be mainly divided into three stages. Stage I: strain is below 0.02, the stress of each material increases linearly with increasing strain in the elastic deformation period of the material. The stress–strain curves with different Co contents show nearly the same tendency. For this stage, the calculated elastic modulus of the WC–12Co cermet is about 446.5 GPa, which is very close to the experimental value of 475 GPa, measured for the nanocrystalline WC–12Co cermet (Wang *et al.*, 2016 ▸). Stage II: strain is between 0.02 and the yield point, and the slope of the stress–strain curve gradually decreases. Moreover, with increasing Co content, the slope of the stress–strain curve and the stress at the yielding point both decrease. Stage III: the yielding period, *i.e.* the stress gradually decreases from the yield point. Occurrences of cracks were found at strains of 0.150, 0.172 and 0.195 for the WC–8Co, WC–12Co and WC–16Co samples, respectively.

### 3.2. Plastic deformation of Co in cermet   

#### 3.2.1. Dislocation density in Co   

**Figure 3:**
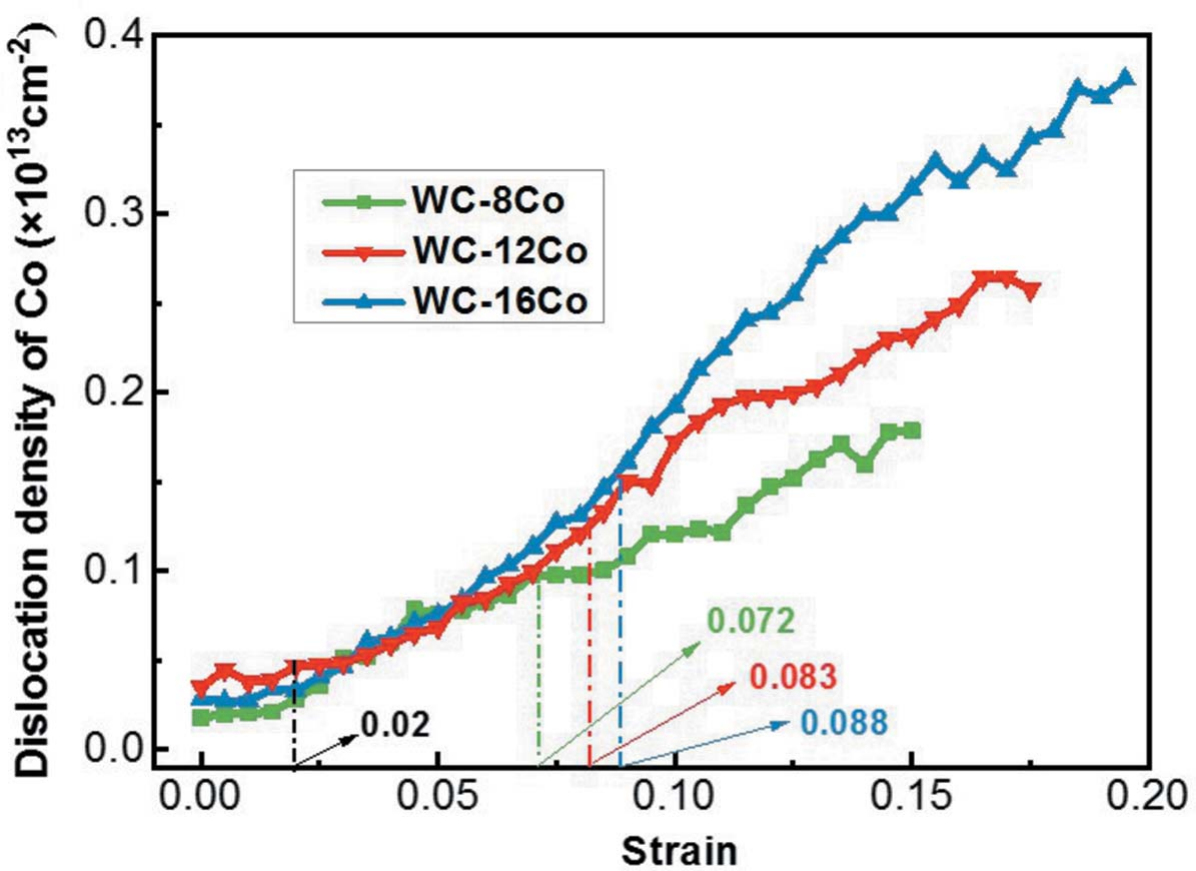
Dislocation densities of Co with strain in the nanocrystalline cermets with different Co contents.

The dislocation densities in Co as a function of strain for the three simulated materials are illustrated in Fig. 3 ▸. There are four periods of Co deformation that can be observed in the curves. In the first period, when the strain is below about 2%, both WC and Co are elastically deformed; during this period no dislocation was generated. In the second period, plastic deformation of Co starts to occur while WC is still at the elastic state. Thus, dislocations are observed only in Co. At this stage, the Co dislocations have similar densities in the three samples.

In the third period, the dislocations in WC initiate. The cermet changes from local plastic deformation (in Co) to overall plastic deformation. In this period the density of Co dislocations increases rapidly with strain in each material, indicating the important contribution of Co to the plastic deformation of the cermet. The strain threshold for the nucleation of WC dislocation increases with the increase of Co content in the cermet. The WC dislocation occurred at strains of 7.2, 8.3 and 8.8% for WC–8Co, WC–12Co and WC–16Co, respectively.

In the fourth period, the growth rate of the Co dislocation density decreases with loading. In addition, this period starts later in the cermet with higher Co content. For example, the growth rate of the dislocation density in Co slowed down at a strain of about 0.135 in the WC–8Co sample, whereas the corresponding strains are 0.165 and 0.185 for the WC–12Co and WC–16Co samples, respectively.

Obviously, in the later periods of deformation, at the same level of strain, the dislocation density in Co increases with the Co content in the cermet. This implies that the dislocations in Co develop faster in the cermet with higher Co content.

#### 3.2.2. Distribution of Co dislocations   

**Figure 4:**
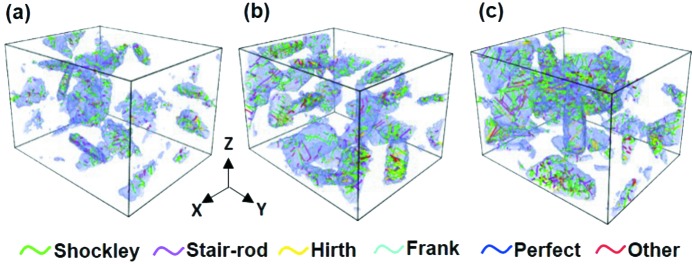
Three-dimensional distributions of Co dislocations in the nanocrystalline cermets with different Co contents at a strain of 0.15 for (*a*) WC–8Co, (*b*) WC–12Co and (*c*) WC–16Co. The purple regions enclose the dislocation cores and surrounding defects in the Co phase.

The aforementioned dislocation activities in Co become straightforward in the three-dimensional distribution of dislocations. Fig. 4 ▸ presents an example of dislocation distributions in Co at a strain of 0.15 for the three samples. The main types of dislocations in Co include Shockley partial dislocations (green), stair-rod dislocations (magenta) and Hirth dislocations (yellow). The dislocation density in Co increases with increasing Co content in the cermet, as shown in Figs. 4 ▸(*a*)–4(*c*). This indicates that Co has a larger contribution to the plastic deformation of the cermet with a higher Co content. This can probably be attributed to the mean free path of Co and the contiguity of WC in the cermet. Table 2 ▸ summarizes the calculated results of the Co mean free path; it increases with the increasing Co content, which is consistent with the experimental findings (Liu *et al.*, 2012 ▸; Luyckx & Love, 2003 ▸)

In contrast, the contiguity of WC grains decreases with the increase of Co content, meaning the WC/Co phase boundaries increase as well. As a result, stress transfer from WC to Co during deformation becomes more significant in the cermet with higher Co content. This leads to a higher degree of plastic deformation of Co. Besides, as the Co content increases in the cermet, the Co mean free path increases and the mean length of the WC/WC grain boundaries decreases. It is thus possible that some of the Co dislocations propagate bypass the local short WC/WC grain boundaries. Consequently, the deformation capacity of Co increases with the long-distance propagation of the dislocations in Co.

**Figure 5:**
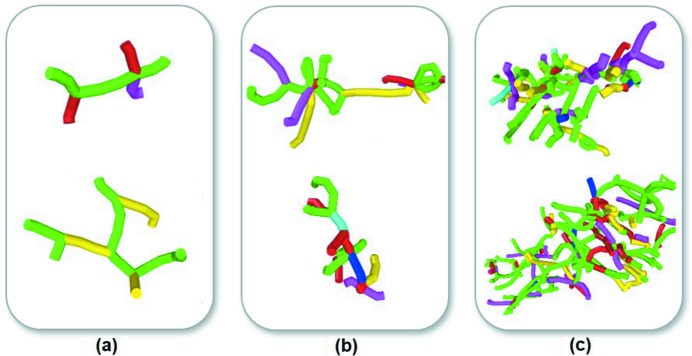
Representative configurations of dislocations at a strain of 0.15 for the cermets with different Co contents: (*a*) WC–8Co, (*b*) WC–12Co, (*c*) WC–16Co.

Furthermore, the local structure and configuration of the dislocations in Co were investigated for the three samples at a strain of 0.15. As shown in Fig. 5 ▸(*a*), in the WC–8Co cermet, the dislocation lines are mainly single and short with a few simple multi-junctions due to the low density. The multi-junction in the dislocation lines locks the mobile dislocations, acting as the obstacle for the dislocation mobility. In the cermets with higher Co content, where the dislocation density is higher, the probability of the dislocation interactions increases. In addition, the structure of the dislocations becomes more complicated at higher Co content in the cermet. Many claw-like multi-junction dislocations started to form in the WC–12Co cermet, as seen in Fig. 5 ▸(*b*). And lots of dislocation tangles and complex dislocation networks were observed in the WC–16Co cermet [Fig. 5 ▸(*c*)] due to the high density of dislocations.

#### 3.2.3. Density of specific dislocations in Co   

**Figure 6:**
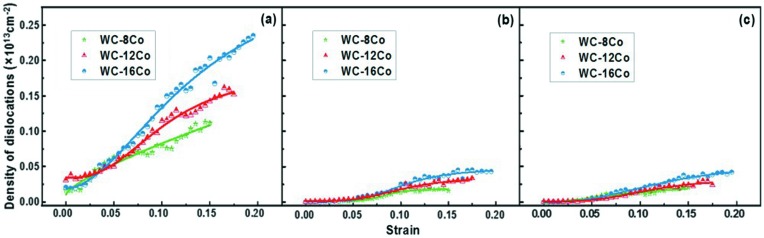
Densities of different Co dislocations as a function of strain and comparison between the different samples: (*a*) Shockley partial dislocations, (*b*) stair-rod dislocations, (*c*) Hirth dislocations.

Fig. 6 ▸ shows the densities of different types of Co dislocations as a function of strain for the simulated samples with different Co contents. As is seen, the Shockley partial dislocation dominates the dislocation density of Co, whereas the other types of dislocations including the stair-rod and Hirth dislocations contribute equally little to the total dislocation density of Co. As the strain increases, the Shockley-type dislocation density increases significantly and a higher dislocation density is observed in the cermet with higher Co content.

#### 3.2.4. Proportions of mobile and sessile dislocations in Co   

**Figure 7:**
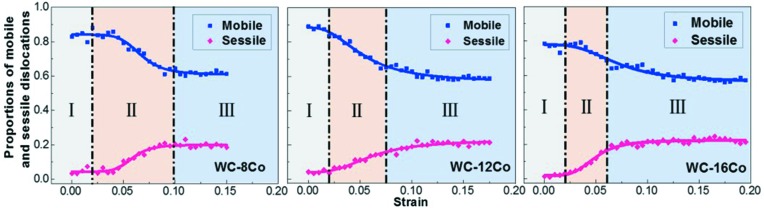
Changes in the proportions of mobile and sessile dislocations in Co of the nanocrystalline cermets with strain for different Co contents.

Fig. 7 ▸ shows the proportions of mobile and sessile dislocations in Co as a function of strain in the nanocrystalline WC–8Co, WC–12Co and WC–16Co cermets. The mobile dislocations are mainly Shockley partial dislocations with Burgers vectors of 1/6〈112〉; they play an important role in plastic deformation of the cermet. The sessile dislocations are mainly Hirth dislocations with Burgers vectors of 1/6〈110〉 and stair-rod dislocations with Burgers vectors of 1/3〈001〉; they are unfavorable to the plastic deformation of Co. The changes in the proportions of mobile and sessile dislocations with strain can be divided into three stages, as marked in Fig. 7 ▸.

**Figure 8:**
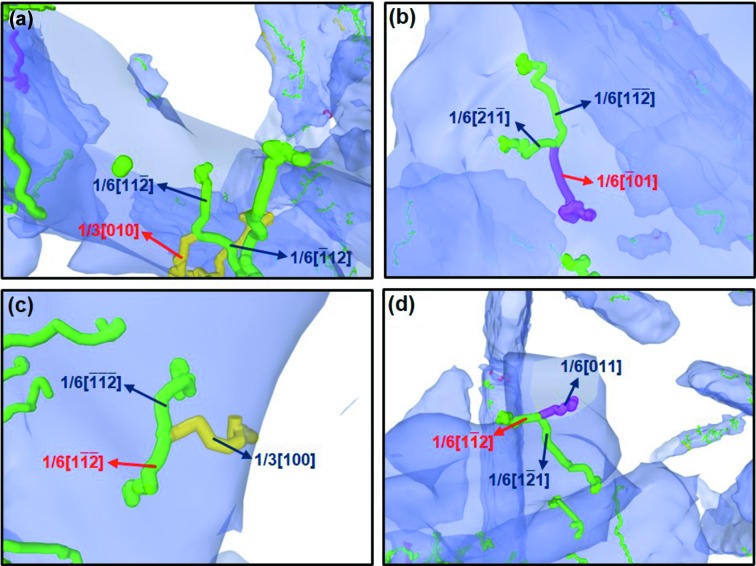
Typical reactions of dislocations in Co, which lead to pinning and depinning effects on dislocations, using WC–8Co as an example: (*a*) reaction of Shockley partial dislocations that leads to the Hirth lock, (*b*) reaction of Shockley partial dislocations that leads to the Lomer–Cottrell lock, (*c*) reaction of Hirth dislocation and Shockley partial dislocation that leads to the Shockley partial dislocation, (*d*) reaction of stair-rod dislocation and Shockley partial dislocation that leads to the Shockley partial dislocation.

At stage I, corresponding to the state of elastic deformation, the proportions of different Co dislocations show little change with strain. At stage II, corresponding to the period of plastic deformation before yielding, the proportions of both mobile and sessile dislocations in Co have a sharp change with strain, indicating pinning and depinning of dislocations may occur in this period. As shown in Figs. 8 ▸(*a*) and 8(*b*), the Shockley partial dislocations were locked due to the reactions, *e.g.* 1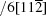 + 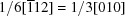 and 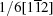 + 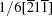 = 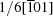. On the other hand, the dislocation locks can be released by the following reactions: 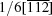 + 1/3[100] = 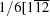] and 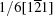 + 1/6[011] = 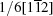, as shown in Figs. 8 ▸(*c*) and 8(*d*). In this period, the proportion of mobile dislocations decreases while that of sessile dislocations increases (see Fig. 7 ▸), implying that dislocation pinning dominates the interaction of dislocations at this stage. At stage III, corresponding to the period of yielding, the dislocation pinning and depinning in Co reaches dynamic equilibrium. The relative proportion of mobile and sessile dislocations became stable, which is about 3:1 at the yielding stage.

Combining Figs. 2 ▸ and 7 ▸, one can see that with the increase of Co content, stage II is narrower and stage III is broadened. This indicates that the cermet with a larger Co content will have a stable yielding state earlier, and this stage remains constant for a long time with increasing strain. The typical reactions of dislocations that lead to dislocation pinning and depinning are similar for the cermets with different Co content, as shown in Fig. 8 ▸.

### 3.3. WC dislocations in cermet   

#### 3.3.1. Nucleation of WC dislocations   

**Figure 9:**
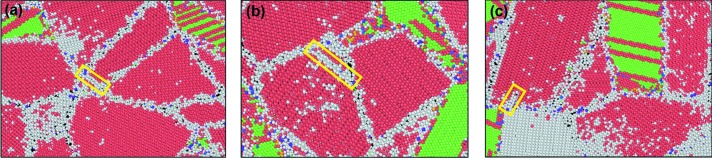
The initiation of WC dislocations in the cermets with different Co contents: (*a*) WC–8Co at a strain of 0.072, (*b*) WC–12Co at a strain of 0.083, (*c*) WC–16Co at a strain of 0.088.

Fig. 9 ▸ shows the atomic snapshots of microstructures in the cermets, which correspond to the nucleation stage of WC dislocations in the samples with different Co contents, respectively. The nucleation of WC dislocations is initiated earlier in the cermet with lower Co content. This is because with a lower Co content, the contiguity of WC grains is larger even though the fraction of WC/Co phase boundaries is lower; thus, the probability that the stress transfers towards Co from WC and WC/Co interface is lower. As a result, the dislocations are generated in WC grains with higher local stress. When the stress concentration cannot be released at the WC grain boundaries, microcracks may occur and expand along the grain boundaries, leading to intergranular fracture of the cermet.

#### 3.3.2. Density of WC dislocations   

**Figure 10:**
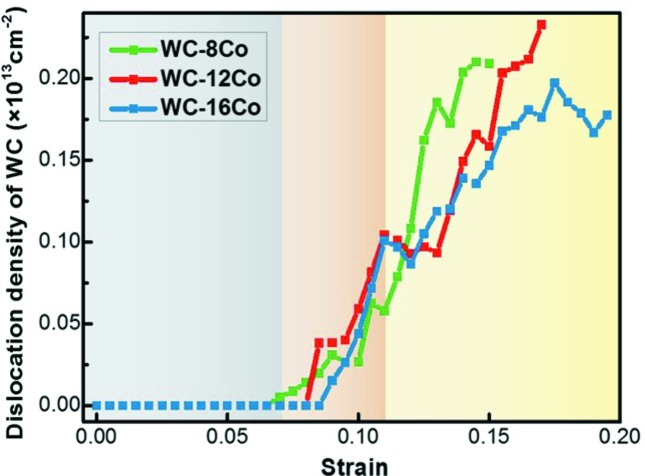
Changes of WC dislocation densities with strain in the nanocrystalline cermets with different Co contents.

The densities of WC dislocations as a function of strain are shown in Fig. 10 ▸ for samples with different Co contents. From the tendency of WC dislocations, the curves can also be divided into three stages, as demonstrated by different background colors in Fig. 10 ▸. At the first stage (gray area), the WC dislocations are not yet activated. In the following stage, WC dislocations initiate in WC–8Co, WC–12Co and WC–16Co at strains of 7.2, 8.3 and 8.8%, respectively. Despite the difference in Co content, the values of WC dislocation density are similar in the three cermet samples. In the third stage, the densities of WC dislocations increase rapidly in the three samples. The growth rate of the WC dislocation density with respect to strain increases with decreasing Co content, which has an opposite tendency to that shown in Fig. 3 ▸.

### 3.4. Contributions of WC and Co to plastic deformation at different stages   

**Figure 11:**
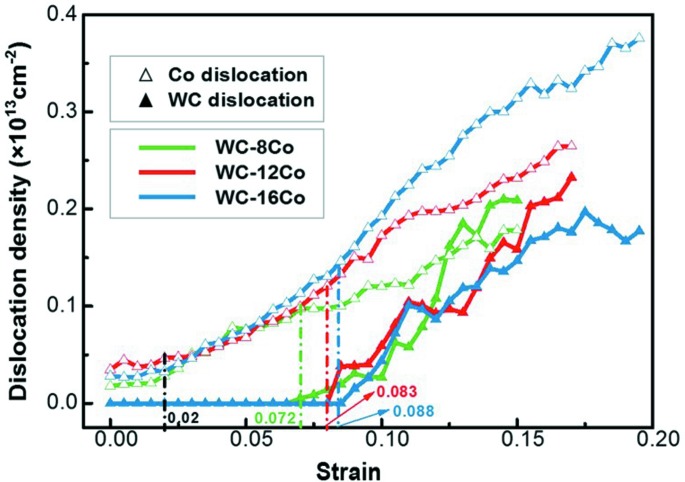
Comparison of WC and Co dislocation densities at different deformation stages for samples with different Co contents.

The dislocation densities of WC and Co were compared for the purpose of analyzing their contributions to the plastic deformation of the cermet at different stages. At the early stage, Co contributes completely to the plastic deformation of the whole material. In the following stage, Co dislocation densities increase rapidly with loading in all the samples. Then, with the rapid increase of the WC dislocation density, the growth rate of the Co dislocation density decreases. Particularly, in the cermet with low Co content, the density of WC dislocations became higher than that of Co dislocations at larger strains, as shown in Fig. 11 ▸. This indicates that in the cermet with low Co content, with loading at stages with large strains WC may contribute more to the plastic deformation of the cermet than Co. However, with a high Co content in the cermet, Co always contributes much more than WC to the plastic deformation. It is worth noting that the contribution of WC to the plastic deformation in the low-Co cermet can be close to that of Co in the medium-Co cermet.

Based on the above comparison, it can be seen that the relative contributions of Co and WC to the plastic deformation of the cermet vary with the deformation stage. This indicates that, in the low-Co cermets when the WC dislocationsinitiate, certain plasticity can be expected for the material, which results from a significant contribution of the WC matrix. It implies that good toughness can also be achieved in the nanocrystalline cermets with low Co contents, if the transgranular fracture is inhibited by modulating dislocations in the WC grains.

## 4. Discussion   

Here the effects of Co content on the deformation features and mechanisms of the nanocrystalline cermet are discussed.

As expected, the cermets with higher Co content have lower yield strength and elastic modulus, which were observed clearly in the stress–strain curves shown in Fig. 2 ▸. Corresponding to the lower yield strength, in the cermet with higher Co content, Co dislocations initiate earlier at a smaller strain. Moreover, the simulation results indicate that the deformation mechanisms are affected by the Co content, *i.e.* the relative contributions of WC and Co dislocations to the plastic deformation of the cermet are influenced by the Co content. This is not a direct effect of the Co percentage, but the initiation of dislocations in different phases and their evolutions with loading.

Co contributes completely to the plastic deformation while WC dislocations do not initiate at smaller strains. At the same strain, the configuration of the Co dislocation lines is more complicated in the cermet with higher Co content. Furthermore, with higher Co content, the ratio of mobile and sessile dislocations in Co becomes stable earlier with a smaller strain. However, the types of dislocations in Co and their proportions appear independent of Co content. For example, the Shockley partial dislocations are distributed widely in Co and make a major contribution to the plastic deformation. Dislocation pinning and depinning both occur in cermets with different Co contents. In all samples, the densities of stair-rod dislocations and Hirth dislocations are close to each other and account for about 1/5 of that of the Shockley partial dislocations. The stair-rod and Hirth dislocations hindered the continuation of the plastic deformation.

For WC, in the cermet with lower Co content, the dislocations were generated at a lower strain, as observed in Fig. 9 ▸, indicating that WC participates in the plastic deformation earlier. Furthermore, the density of WC dislocations gradually increases to a certain extent, and with the interaction of dislocations, tangles and reactions of dislocations may occur, leading to sessile dislocations and work hardening in WC. The stress concentration may result in the nucleation of microcracks. This could be the reason why the cermet with lower Co content is prone to fracture earlier.

## 10. Conclusions   

In this work, the mechanical behavior and deformation mechanisms of nanocrystalline cermets with different Co content were studied by MD simulations. The following conclusions were made:

(i) The content of Co clearly affects the mechanical properties of the nanocrystalline cermets through modulating the deformation process. With higher Co content, the ratio between mobile and sessile dislocations in Co becomes stable earlier in the cermet. However, this ratio stabilizes to a value of 3:1 at the final stage of deformation regardless of Co content, owing to the dynamic equilibrium between pinning and depinning of Co dislocations through reactions.

(ii) The Shockley partial dislocations are distributed widely in Co and make a major contribution to the plastic deformation. Independent of Co content, the densities of the stair-rod and Hirth dislocations are close to each other and count for about 1/5 of that of the Shockley partial dislocations. The stair-rod and Hirth dislocations of Co hinder the continuation of plastic deformation of the cermet.

(iii) Nucleation of dislocation in WC has a smaller strain threshold in the cermet with lower Co content. Moreover, in the low-Co cermet, the growth rate of WC dislocation density increases rapidly at the late stage of deformation, which shows an opposite tendency to that of Co dislocation density. As a result, the density of WC dislocations may be higher than that of Co dislocations at larger strains in the cermet with lower Co content.

(iv) Co is responsible for the plasticity of the cermet at the beginning of the plastic deformation. As WC starts to participate in the plastic deformation of the cermet, its dislocation density is much lower compared with that of Co. However, with loading the relative contribution of Co and WC to the plastic deformation of the cermet varies with the deformation stage. With the WC dislocation density exceeding the Co dislocation density in the low-Co cermet, WC plays a more important role in the plasticity of the cermet at the later stage of deformation.

## 6. Conflict of interest statement   

The authors declare no conflict of interest.

## Figures and Tables

**Table 1:** Related parameters of the simulated nanocrystalline cermets

Designed composition	WC–8Co	WC–12Co	WC–16Co
Real Co content (%)	7.78	12.05	16.06
Mean WC grain size (nm)	13.21	13.57	13.51
Model dimension (nm)	28.9×35.2×28.9	28.9×35.3×28.9	28.9×35.4×28.9
Number of atoms	2832774	2821128	2820384

**Table 2:** WC grain contiguity and Co mean free path in the simulated nanocrystalline cermets

	WC grain contiguity	Co mean free path (nm)
WC–8Co	0.7727	1.355
WC–12Co	0.7103	1.643
WC–16Co	0.636	1.881
